# Tissue clock-guided prediction and intervention of futile recanalization: towards precision therapy in mechanical thrombectomy

**DOI:** 10.3389/fneur.2026.1768821

**Published:** 2026-04-09

**Authors:** Yuexin Wu, Baoyan Liu, Jian Ma, Xianxian Zhao

**Affiliations:** Department of Emergency Medicine, Binzhou People's Hospital, Binzhou, Shandong, China

**Keywords:** futile recanalization, individualized prediction, mechanical thrombectomy, precision intervention, stroke, tissue clock

## Abstract

Mechanical thrombectomy (MT) is the standard of care for acute ischemic stroke caused by large vessel occlusion (LVO). Yet despite achieving high rates of angiographic reperfusion, nearly half of treated patients do not regain functional independence—a phenomenon termed futile recanalization (FR). This persistent gap between vessel patency and clinical recovery exposes the fundamental limitations of traditional time-based treatment paradigms, which assume a uniform rate of ischemic progression across individuals. The pathophysiology of FR is multifactorial, involving microvascular no-reflow, early arterial reocclusion, collateral circulation failure, and reperfusion-mediated injury. These mechanisms interact in a complex, temporally evolving cascade that cannot be captured by a single imaging or clinical metric. The emerging “tissue clock” framework reframes patient selection from elapsed time to individualized tissue viability, drawing on advanced imaging biomarkers including diffusion–FLAIR mismatch, net water uptake quantification, infarct core–penumbra dynamics, and collateral hemodynamic assessment. The DAWN and DEFUSE 3 trials provided landmark evidence that imaging-guided selection enables safe and effective thrombectomy well beyond conventional time windows, validating the clinical relevance of tissue-based decision-making. In parallel, predictive modeling has evolved from traditional clinical scoring systems toward machine learning–based and multimodal approaches that integrate clinical, imaging, and biological variables for individualized risk stratification. The tissue clock paradigm thus marks a conceptual shift from population-level time thresholds to individualized pathophysiological assessment. By integrating imaging biomarkers, circulating biological indicators, and computational prediction models, clinicians may achieve more accurate outcome prediction and deploy multi-target interventions to mitigate FR. Realizing this vision will require standardized tissue clock quantification protocols, prospective validation of artificial intelligence models across diverse populations, and translational evaluation of combination therapies—ultimately aligning successful recanalization with durable functional recovery.

## 1 Introduction

Acute ischemic stroke remains a leading cause of adult disability and mortality worldwide. Large vessel occlusion (LVO) accounts for 30–50% of all ischemic strokes, yet it is responsible for a disproportionate share of severe neurological deficits and long-term dependence (1, 2). The treatment landscape for LVO stroke was transformed in 2015, when five landmark randomized controlled trials demonstrated that mechanical thrombectomy (MT) within 6 h of symptom onset significantly improves functional outcomes compared with medical therapy alone (3–5). Subsequently, the DAWN and DEFUSE 3 trials extended the treatment window to 16–24 h by employing advanced imaging to select patients with favorable tissue profiles (6, 7). Together, these trials established MT as the standard treatment for LVO and reshaped the organizational framework of acute stroke care.

**Figure 1:**
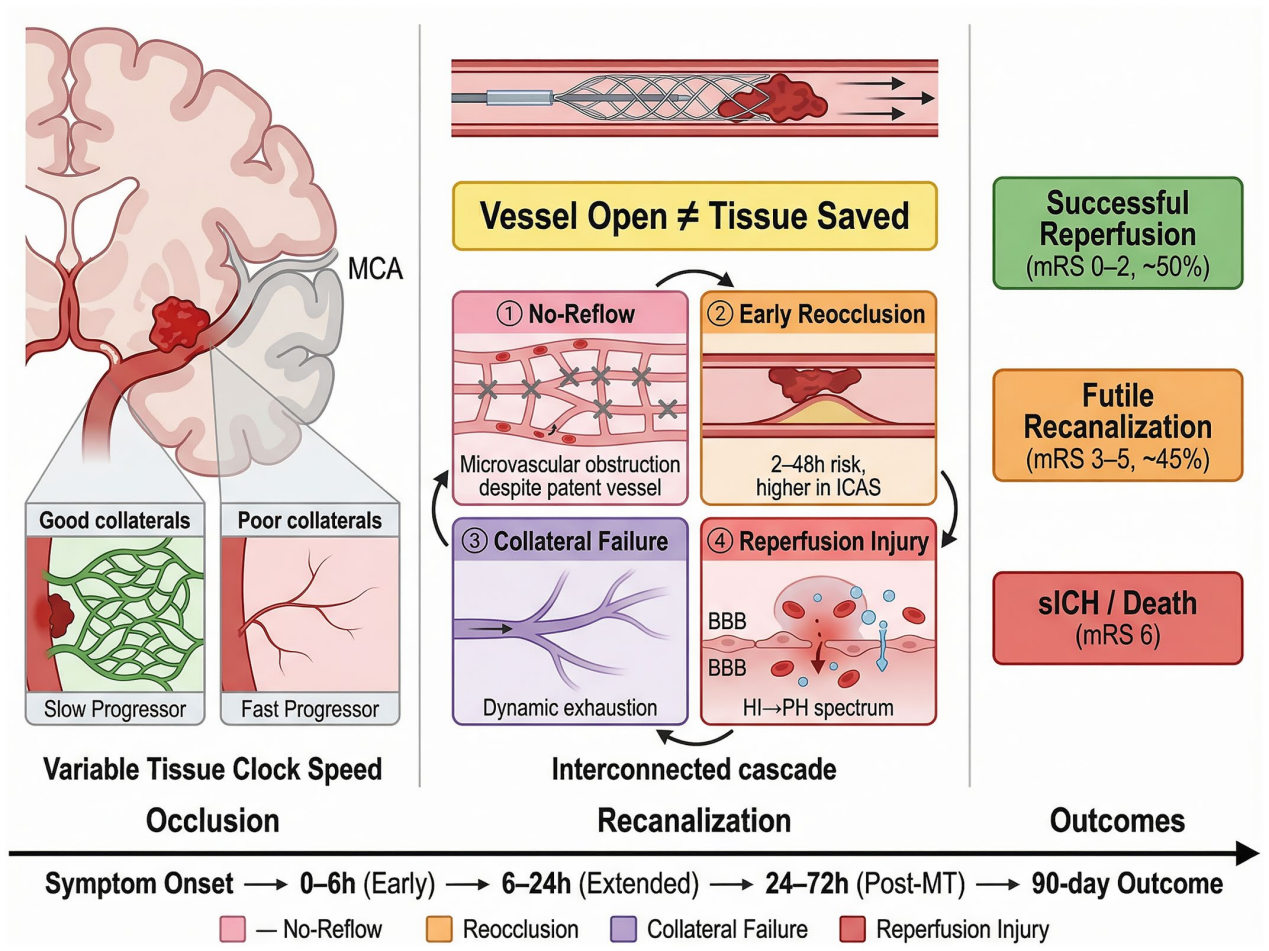
Pathophysiological mechanisms of futile recanalization. Schematic illustration of the dissociation between successful macrovascular recanalization and tissue-level reperfusion failure. The left panel depicts large vessel occlusion with variable collateral circulation status—graded using the ASITN/SIR scale (grade 0, absent collaterals; through grade 4, complete rapid collateral filling) or single-phase CTA Tan score (0–2)—determining tissue clock speed and categorizing patients as slow versus fast progressors. The center panel illustrates four interconnected mechanisms underlying futile recanalization: ① microvascular no-reflow, driven by endothelial swelling, leukocyte adhesion (P-selectin, ICAM-1 mediated), pericyte contraction, and microthrombus formation, resulting in persistent perfusion deficits in 20–40% of recanalized patients; ② early arterial reocclusion (incidence 2–9% overall, up to 18% in ICAS-related occlusions), occurring through endothelial injury, plaque disruption, and residual thrombus; ③ collateral circulation failure, reflecting progressive exhaustion of vasodilatory reserve and hemodynamic decompensation; and ④ reperfusion injury with hemorrhagic transformation classified by the ECASS criteria (HI type 1, petechiae along infarct margins; HI type 2, confluent petechiae; PH type 1, hematoma ≤30% of infarct zone; PH type 2, dense hematoma >30% with mass effect), with symptomatic intracranial hemorrhage elevating futile recanalization risk approximately 7-fold. The right panel shows clinical outcome distribution after successful recanalization: functional independence (mRS 0–2, ~50%), futile recanalization (mRS 3–5, ~45%), and mortality (mRS 6). The bottom timeline indicates temporal progression from symptom onset through the 90-day outcome assessment. Abbreviations: MCA, middle cerebral artery; ICAS, intracranial atherosclerotic stenosis; BBB, blood–brain barrier; ECASS, European Cooperative Acute Stroke Study; HI, hemorrhagic infarction; PH, parenchymal hematoma; sICH, symptomatic intracranial hemorrhage; mRS, modified Rankin Scale.

Against this background of therapeutic progress, a sobering clinical reality persists: despite successful large vessel recanalization—defined as modified Thrombolysis in Cerebral Infarction (mTICI) grade 2b or 3—approximately half of treated patients fail to achieve functional independence at 90 days (8, 9). This phenomenon, termed futile recanalization (FR), is conventionally defined as achieving adequate vessel patency (mTICI ≥2b) while retaining a modified Rankin Scale (mRS) score greater than 2 at the 90-day follow-up assessment (10). The high incidence of FR not only diminishes the net therapeutic benefit of MT at a population level but also imposes substantial burdens on healthcare systems and complicates shared decision-making between clinicians and families (Figure 1).

**Figure 2:**
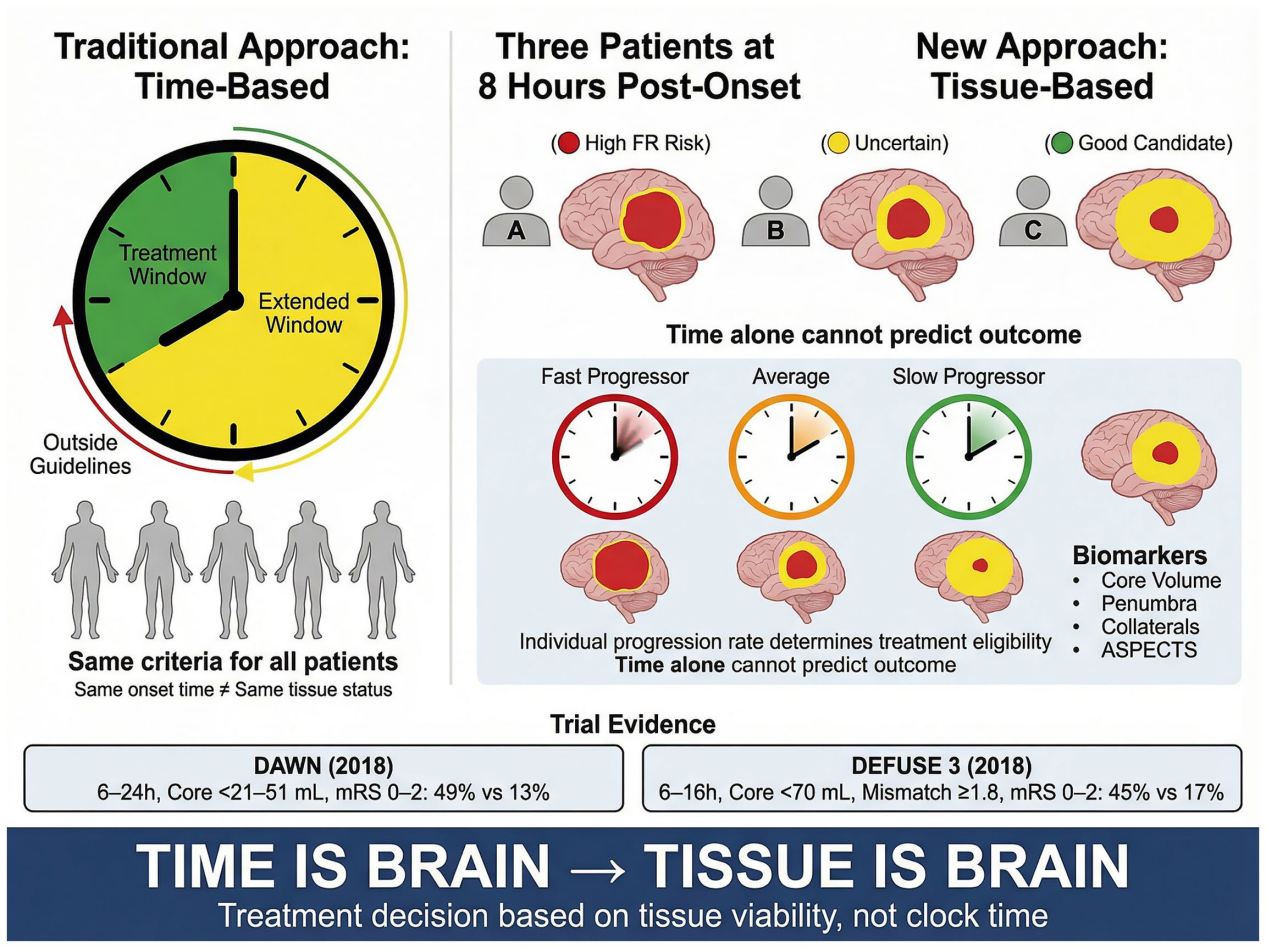
Tissue clock vs. time clock: a paradigm shift in patient selection. Conceptual comparison of time-based and tissue-based treatment selection paradigms. The left panel depicts the conventional time clock approach with fixed treatment windows (0–6 h, early window; 6–24 h, extended window per DAWN/DEFUSE 3 criteria) applied uniformly regardless of individual tissue status. The center panel demonstrates that three patients at an identical 8-h timepoint may exhibit vastly different tissue profiles: patient A, with poor collateral circulation (ASITN/SIR grade 0–1, Tan score 0) and a large infarct core (ASPECTS ≤5, core volume >70 mL), faces high futile recanalization risk; patient B, with moderate collaterals (ASITN/SIR grade 2) and intermediate tissue status, occupies an uncertain treatment zone; patient C, with robust collaterals (ASITN/SIR grade 3–4, Tan score 2) and a small core with favorable mismatch (core <31 mL, mismatch ratio ≥1.8, mismatch volume ≥15 mL), remains an excellent treatment candidate. The right panel illustrates the tissue clock concept: individual tissue progression rates—fast (core growth >10 mL/h), average, and slow (minimal core growth over hours)—determine treatment eligibility based on imaging biomarkers rather than elapsed time. The bottom section summarizes landmark trial evidence: DAWN (6–24 h, clinical-core mismatch; functional independence 49% vs. 13%) and DEFUSE 3 (6–16 h, perfusion mismatch; functional independence 45% vs. 17%), along with key tissue clock biomarkers (ASPECTS, core volume, perfusion mismatch, net water uptake, collateral status, DWI-FLAIR mismatch). Abbreviations: FR, futile recanalization; ASPECTS, Alberta Stroke Program Early CT Score; ASITN/SIR, American Society of Interventional and Therapeutic Neuroradiology/Society of Interventional Radiology collateral grading scale.

The traditional axiom “time is brain” has guided acute stroke treatment for decades (11), resting on the implicit assumption that ischemic injury progresses at a roughly uniform rate across all patients. Accumulating evidence, however, reveals that the tempo of ischemic evolution varies substantially between individuals. This recognition has given rise to the “tissue clock” concept, which holds that tissue viability—rather than symptom onset time alone—more accurately reflects the volume of salvageable brain parenchyma at any given moment (12, 13). At one extreme, “fast progressors” may sustain irreversible infarction within minutes of onset and derive limited benefit even from early recanalization; at the other, “slow progressors” with robust collateral circulation may retain a substantial ischemic penumbra for many hours, remaining candidates for effective intervention well beyond conventional time windows. The DAWN and DEFUSE 3 trials operationalized this concept by selecting patients with a “small core, large penumbra” profile using perfusion or clinical-core mismatch criteria, thereby demonstrating safe and effective late-window thrombectomy (6, 7). These results catalyzed a paradigm shift in acute stroke treatment—from a “time-oriented” to a “tissue-oriented” approach (Figure 2).

**Figure 3:**
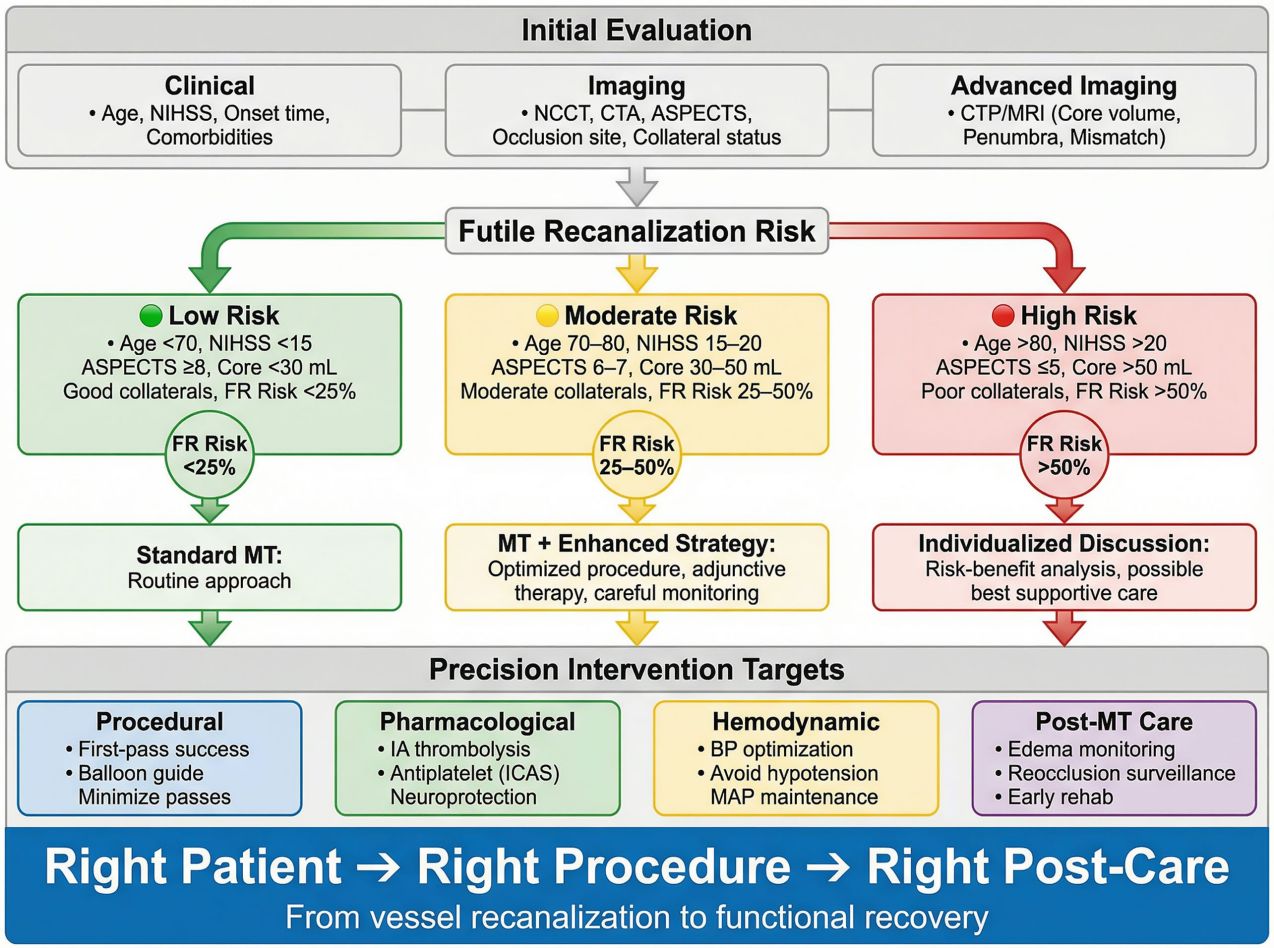
Clinical decision framework for individualized prediction and intervention. Flowchart illustrating a precision medicine approach to futile recanalization risk management across the thrombectomy treatment chain. Initial evaluation integrates clinical parameters (age, baseline NIHSS, comorbidity burden including diabetes and atrial fibrillation, pre-stroke functional status), standard imaging (NCCT for ASPECTS scoring, CTA for occlusion site confirmation and collateral grading using ASITN/SIR or multiphase CTA scales), and advanced perfusion imaging (CTP or MR perfusion for core volume [CBF < 30%], penumbra volume [Tmax >6 s], mismatch ratio, and hypoperfusion intensity ratio [HIR]). Composite risk stratification categorizes patients into low (<25% estimated FR risk; favorable tissue profile with small core, good collaterals, and favorable mismatch), moderate (25–50%; intermediate tissue status with competing favorable and unfavorable features), or high (>50%; large established core [>70 mL or ASPECTS ≤5], poor collaterals [ASITN/SIR 0–1], elevated net water uptake [>10–12%], or advanced age with significant comorbidity burden) futile recanalization risk. Treatment pathways include: for low-risk patients, standard mechanical thrombectomy with routine peri-procedural care; for moderate-risk patients, enhanced procedural strategy (balloon guide catheter use, first-pass optimization, combined aspiration–stent retriever technique) with adjunctive pharmacotherapy (intra-arterial alteplase at 0.225 mg/kg for microvascular thrombi, etiology-guided antiplatelet therapy for ICAS-related occlusions); for high-risk patients, individualized risk–benefit discussion incorporating patient values, with consideration of treatment in selected subgroups (younger age, residual collateral supply, anticipated rapid high-quality recanalization) based on evidence from large-core trials (RESCUE-Japan LIMIT, SELECT 2, ANGEL-ASPECT). The bottom panel outlines four precision intervention domains: ① procedural optimization (first-pass effect targeting, balloon guide catheter, device individualization), ② pharmacological adjuncts (IA thrombolysis, GP IIb/IIIa inhibitors for ICAS, immunomodulators), ③ hemodynamic management (pre-recanalization SBP > 140 mmHg, post-recanalization target 140–180 mmHg, individualized adjustment for large infarcts), and ④ post-thrombectomy neurocritical care (serial neurological monitoring every 1–2 h, reocclusion surveillance, malignant edema management per DECIMAL/DESTINY/HAMLET evidence, glycemic/temperature/seizure control protocols). Abbreviations: MT, mechanical thrombectomy; NIHSS, National Institutes of Health Stroke Scale; NCCT, non-contrast computed tomography; CTA, computed tomography angiography; CTP, computed tomography perfusion; MRI, magnetic resonance imaging; CBF, cerebral blood flow; IA, intra-arterial; ICAS, intracranial atherosclerotic stenosis; BGC, balloon guide catheter; MAP, mean arterial pressure; SBP, systolic blood pressure.

Despite these advances, critical knowledge gaps remain. Current understanding of FR is heavily weighted toward imaging-based prediction, with comparatively limited insight into the complex interplay of its underlying pathophysiological mechanisms—including microcirculatory obstruction, endothelial dysfunction, and individual variation in ischemic vulnerability. Clinical practice still lacks reliable, externally validated risk stratification tools and evidence-based intervention strategies that can be deployed at the bedside. As machine learning and artificial intelligence continue to advance, a pressing challenge is how to integrate multimodal data streams—spanning imaging, laboratory biomarkers, and clinical variables—to construct individualized prediction models, and how to translate the tissue clock concept into actionable prevention and intervention strategies (Figure 3).

This review examines futile recanalization from three complementary perspectives: epidemiology, mechanism, and intervention. In doing so, we treat the tissue clock not simply as a surrogate for elapsed ischemic time, but as an integrated estimate of whether recanalization can still be translated into tissue salvage and meaningful functional recovery. This requires attention not only to infarct core–penumbra mismatch, but also to factors that are less fully captured by conventional time-window thinking, including pre-existing irreversible ischemic injury, lesion topology within eloquent or network-critical regions, and the patient’s biological capacity for recovery. By linking these dimensions to mechanical thrombectomy decision-making and post-reperfusion care, we aim to outline a more operative framework for guiding clinical decisions (Table 1).

**Table 1:** Integrated framework for predicting and mitigating futile recanalization under the tissue clock paradigm.

Domain	Key determinants/indicators	Contribution to futile recanalization	Tissue clock implication	Potential clinical response
Baseline biological vulnerability and recovery reserve	Advanced age, frailty, pre-stroke disability, cerebral small-vessel disease, white matter injury, brain atrophy, major comorbidities	Limits neuroplasticity and functional recovery even after technically successful reperfusion	Viability alone may overestimate the likelihood of functional gain if biological reserve is poor	Incorporate baseline vulnerability into individualized risk assessment and shared decision-making
Pre-treatment ischemic injury	Low ASPECTS, large core volume, severe edema/NWU elevation, poor mismatch profile	A substantial portion of tissue may already be irreversibly injured before thrombectomy, reducing the chance that recanalization translates into benefit	Tissue that is biologically advanced despite a favorable chronological time window calls for imaging-based selection over reliance on onset-to-treatment time alone	Use multimodal imaging to refine selection and avoid relying on onset-to-treatment time alone
Collateral and perfusion status	Collateral grade, HIR, core growth rate, perfusion-defined penumbra	Determines tissue survival before reperfusion and influences progression speed from salvageable penumbra to infarction	How fast the tissue clock runs depends heavily on collateral status	Prioritize rapid reperfusion in fast progressors and use collateral-aware patient selection
Lesion topology and eloquent-region involvement	Internal capsule, thalamus, brainstem, dominant hemispheric language areas, strategic cortical/subcortical network hubs	Small or moderate infarcts in critical locations may still produce severe lasting disability despite recanalization	Volume alone may underestimate functional risk when lesion location is strategically unfavorable	Estimate expected benefit by weighing lesion size against topographic and network-level factors
Tissue-level reperfusion failure	Microvascular no-reflow, distal embolization, capillary obstruction, incomplete downstream perfusion despite mTICI 2b–3	Creates dissociation between macrovascular reopening and effective tissue reperfusion	Angiographic success may mask an unfavorable tissue clock state	Aim for near-complete reperfusion and consider strategies that improve first-pass success and distal perfusion
Procedural factors during mechanical thrombectomy	First-pass effect, number of passes, BGC use, aspiration vs. stent retriever vs. combined technique, procedure duration	Repeated manipulation may increase endothelial injury, distal embolization, and reperfusion failure	Procedural execution determines how much viable tissue is ultimately salvaged	Optimize device strategy, pursue first-pass near-complete reperfusion, and minimize unnecessary passes
Post-reperfusion secondary injury	Reperfusion injury, hemorrhagic transformation, blood–brain barrier disruption, inflammation, early arterial reocclusion	Secondary deterioration may negate the arterial benefit of successful recanalization	Reperfusion does not stop the tissue clock; without protective care, secondary injury may continue to advance it	Intensive post-thrombectomy monitoring, blood pressure control, and mechanism-guided adjunctive therapy
Integrated prediction and precision management	Clinical scores, multimodal imaging, ML/deep learning models, circulating biomarkers	Single variables incompletely capture FR risk; integrated models better represent patient heterogeneity	The tissue clock is best understood as a multidimensional construct that requires continuous updating	Develop interpretable, multimodal, and bedside-ready tools for individualized treatment planning

ASPECTS, Alberta Stroke Program Early CT Score; NWU, net water uptake; HIR, hypoperfusion intensity ratio; mTICI, modified Thrombolysis in Cerebral Infarction; BGC, balloon guide catheter; ML, machine learning; FR, futile recanalization.

## 2 Epidemiological characteristics of futile recanalization

### 2.1 Definition and incidence

The central paradox of futile recanalization lies in the failure of successful vessel reopening to produce clinical benefit. The most widely adopted definition requires angiographic reperfusion of mTICI 2b or 3—indicating substantial or complete restoration of antegrade flow—coupled with a 90-day mRS score of 3 or greater, signifying failure to achieve functional independence (14). Although this dichotomous classification provides a practical framework for clinical research, it compresses a heterogeneous spectrum of outcomes into a single category: the patient who dies (mRS 6) and the patient with moderate disability requiring some assistance (mRS 3) are counted identically, despite vastly different clinical trajectories and implications for care. Moreover, a fixed 90-day assessment point may underestimate the potential for delayed neurological recovery, particularly in younger patients or those with posterior circulation strokes, where functional gains may continue beyond 3 months (15).

The reported incidence of FR varies considerably across studies, driven by differences in inclusion criteria, recanalization definitions, and follow-up protocols. A comprehensive meta-analysis estimated a pooled FR incidence of approximately 51% (95% CI: 48–54%) (8), while the HERMES individual patient data meta-analysis—aggregating five major thrombectomy trials—reported that roughly 43% of patients with successful recanalization still experienced FR (3). These discrepancies reflect not only methodological heterogeneity but also variation in the patient populations studied: trials with strict imaging-based selection tend to report lower FR rates than observational registries with broader enrollment (16). An encouraging temporal trend has emerged, however. In studies published between 2010 and 2015, FR rates commonly exceeded 60%, whereas more recent cohorts—benefiting from improved patient selection, refined thrombectomy techniques, and faster workflow protocols—report rates closer to 40% (17). Even so, a rate approaching one in two treated patients underscores the magnitude of unmet clinical need.

### 2.2 Risk factors

The risk of FR is shaped by the convergence of patient-level vulnerability, imaging characteristics at presentation, and procedural variables. Among patient-related factors, age is the most consistently identified predictor. Meta-analytic data indicate that each 10-year increment in age is associated with an approximately 30% increase in FR risk (8), though this relationship is not strictly linear: the inflection point appears to lie between 75 and 80 years, beyond which the marginal increase in risk accelerates. Of note, the negative prognostic impact of advancing age can be partially attenuated by favorable collateral circulation, suggesting that biological rather than chronological age may be more relevant to treatment decisions (3, 18). Baseline stroke severity, measured by the National Institutes of Health Stroke Scale (NIHSS), exhibits a parallel association: patients presenting with NIHSS scores exceeding 20 face substantially elevated FR risk. The NIHSS, however, has recognized limitations in posterior circulation strokes and non-dominant hemisphere infarctions, where clinically significant deficits may not be fully captured by the scale (19, 20).

Comorbid conditions further modulate FR risk through distinct pathophysiological pathways. Diabetes mellitus increases the likelihood of FR (OR 1.4; 95% CI: 1.2–1.7), an effect attributed to chronic microvascular endotheliopathy, impaired autoregulation, and enhanced susceptibility to ischemia–reperfusion injury (21). The influence of hypertension is more nuanced: chronic hypertension may promote arteriogenesis and thus enhance collateral capacity, yet acute hypertension in the peri-procedural period elevates the risk of hemorrhagic transformation (22). The so-called “smoking paradox”—whereby some observational analyses report a lower FR incidence among smokers—has been largely attributed to selection bias and confounding rather than a genuine protective effect.

Beyond conventional vascular risk factors, diminished recovery reserve may also help explain why some patients experience FR despite technically successful reperfusion. Advanced age, frailty, cerebral small-vessel disease, pre-existing white matter injury, and brain atrophy may reduce network resilience and limit post-stroke neuroplasticity, thereby weakening the functional benefit that can be achieved even when arterial patency is restored. These factors, however, do not always manifest as a larger infarct core at presentation; rather, they may constrain the brain’s ability to convert tissue survival into neurological recovery. This distinction matters in practice: FR risk may reflect not only the extent of acute ischemic injury, but also the pre-morbid capacity for functional restoration.

Imaging-based predictors occupy a central position in FR risk stratification. The Alberta Stroke Program Early CT Score (ASPECTS) provides a standardized, semiquantitative assessment of early ischemic changes on noncontrast CT across 10 defined regions of the middle cerebral artery territory; lower scores indicate more extensive early injury and correlate with higher FR risk (23). In practice, however, ASPECTS suffers from moderate interrater reliability (*κ* = 0.6–0.7), which falls further among non-specialist readers such as emergency physicians (24). Automated scoring tools (RAPID ASPECTS, e-ASPECTS) achieve agreement with expert neuroradiologists in the range of 0.7–0.8, partially mitigating subjectivity but introducing concerns about dependence on image quality and cross-platform generalizability (25, 26). Collateral circulation status, most commonly assessed by single-phase CT angiography (CTA) using scales such as the Tan score or ASITN/SIR grading system, is recognized as a powerful prognostic indicator. Single-phase CTA, however, provides only a single temporal snapshot of what is an inherently dynamic hemodynamic process; multiphase CTA addresses this limitation by acquiring images at multiple time points, enabling differentiation of rapidly and slowly filling collateral pathways, though at the cost of increased radiation exposure and contrast agent burden (27, 28). Fundamentally, the causal relationship between collateral adequacy and FR has not been firmly established—it remains uncertain whether poor collaterals directly cause FR or merely serve as a marker of more advanced, irreversible ischemic injury (29). Perfusion imaging, which defines the ischemic core (commonly as tissue with cerebral blood flow <30% of normal) and penumbra (Tmax >6 s), is central to tissue clock-based decision-making. The thresholds employed by the most widely validated platform, RAPID software, were derived from specific study populations and may not be universally applicable across different stroke etiologies and patient demographics (30–32).

Procedural variables represent a modifiable domain of FR risk. Time to recanalization remains a critical determinant: each one-hour delay in achieving reperfusion is associated with an approximately 15% increase in FR risk, although the magnitude of this effect attenuates in later time windows, consistent with the concept that patients eligible for late treatment have inherently slower tissue progression (33). Notably, in-hospital process delays—particularly the interval from door to arterial puncture—often contribute more to unfavorable outcomes than does the total elapsed time from symptom onset, highlighting the importance of institutional workflow optimization (34). Technical aspects of the thrombectomy procedure are also significantly associated with FR. Use of a balloon guide catheter, achievement of complete recanalization on the first device pass (the first-pass effect), and minimization of total thrombectomy attempts have each been independently linked to better outcomes (35). These technical factors, however, do not operate in isolation: in patients with poor collateral circulation, even rapid and technically successful recanalization may prove insufficient to salvage tissue that has already progressed beyond the threshold of reversibility (36).

### 2.3 Methodological limitations and implications for future research

Several methodological constraints limit the conclusions that can be drawn from current FR epidemiology. The mRS, while practical and widely adopted, is an ordinal scale with coarse granularity that cannot capture subtle but clinically meaningful functional differences; a single 90-day assessment may further obscure ongoing recovery trajectories (15). The evidence base is predominantly drawn from high-volume academic stroke centers, where case selection, operator experience, and ancillary care may differ substantially from community practice settings; real-world FR rates in less specialized environments are likely higher (37). Additionally, anterior and posterior circulation strokes, proximal and distal occlusions, and strokes of different etiological subtypes (cardioembolic, atherosclerotic, dissection-related) likely carry distinct FR risk profiles that are obscured when pooled in aggregate analyses (20). Most existing prediction models have been developed using single-center retrospective cohorts and lack rigorous external validation across diverse populations and healthcare systems (38).

Taken together, these limitations indicate that the epidemiological understanding of FR must evolve beyond broad risk factor attribution. Advancing toward refined, individualized prediction will require integration of granular patient-level clinical data, quantitative imaging biomarkers, and treatment process metrics within analytic frameworks capable of capturing their complex interactions.

## 3 Pathophysiological mechanisms of futile recanalization

### 3.1 Overview of the recanalization–reperfusion dissociation

The core pathophysiology underlying futile recanalization is the dissociation between macrovascular recanalization and meaningful tissue and functional recovery. Reopening an occluded large artery does not guarantee that blood flow will adequately reach the downstream capillary bed, nor does restored flow necessarily imply that sufficient viable tissue remains to support neurological improvement. In some patients—particularly fast progressors or those with poor collateral support—a substantial portion of the affected territory may have already crossed the threshold of irreversible ischemic injury before thrombectomy is performed. In others, the final clinical outcome is disproportionately shaped by lesion topology: relatively limited infarction involving eloquent structures or network hub regions, such as the internal capsule, thalamus, brainstem, or strategic cortical association areas, may still result in severe persistent disability despite angiographic success (39). Compounding this, four highly interconnected processes—microvascular no-reflow, early arterial reocclusion, collateral circulation failure, and reperfusion-mediated injury—further widen the gap between arterial reopening and functional recovery. These processes do not act in isolation; instead, they interact across temporal and spatial scales to form a self-reinforcing pathological cascade (9). This broader dissociation helps explain the heterogeneity of outcomes after thrombectomy and points toward rational targets for intervention.

### 3.2 Microvascular no-reflow

The no-reflow phenomenon—first described by Ames and colleagues in 1968 using a rabbit model of global cerebral ischemia—refers to the persistent failure of microcirculatory perfusion despite adequate restoration of proximal arterial patency (40). At the molecular and cellular level, several converging mechanisms contribute to capillary obstruction. Endothelial cells swell in response to ischemic energy failure and loss of ion homeostasis, physically narrowing the capillary lumen. Simultaneously, activated leukocytes—particularly neutrophils—adhere to the endothelium via upregulated adhesion molecules (P-selectin, ICAM-1) and become trapped within capillaries whose caliber (5–8 μm) is comparable to the diameter of a deformed leukocyte. Microthrombi composed of platelets, fibrin, and cellular debris further impede flow. At the level of the neurovascular unit, pericytes—contractile cells that ensheath capillaries—constrict irreversibly in response to oxidative and nitrosative stress, maintaining elevated microvascular resistance even after upstream recanalization (41). Astrocytic endfeet swelling adds an additional compressive force on the abluminal surface of capillaries (42).

Clinical imaging studies suggest that 20–40% of patients who achieve successful macrovascular recanalization exhibit persistent perfusion deficits on follow-up imaging, indicative of incomplete microcirculatory reperfusion (39). The true prevalence, however, is uncertain: estimates vary depending on the imaging modality employed, the time interval between recanalization and assessment, and the thresholds used to define hypoperfusion. Current clinical imaging techniques—including CT perfusion and MR perfusion—lack the spatial resolution to directly visualize capillary-level flow obstruction, and it remains debated whether “no-reflow” should be defined as complete absence of microvascular flow or as a significant quantitative reduction below a functional threshold. Among candidate predictive biomarkers, larger infarct core volume, elevated peripheral white blood cell count, and admission hyperglycemia have each been associated with increased no-reflow risk, though their individual predictive accuracy is insufficient for clinical decision-making in isolation (43). Integrating multiple biomarker categories—including inflammatory mediators (C-reactive protein, matrix metalloproteinases), endothelial activation markers (von Willebrand factor, thrombomodulin), and real-time perfusion parameters derived from advanced imaging—may enhance the ability to identify patients at highest risk for this complication before or immediately after thrombectomy (44).

### 3.3 Early arterial reocclusion

Early arterial reocclusion (EAR) refers to the re-establishment of arterial occlusion at or near the original site within hours to days after initially successful thrombectomy. The reported incidence ranges from 2 to 9% in general stroke populations, but rises to approximately 18% in patients whose occlusion is attributable to intracranial atherosclerotic stenosis (ICAS), where an underlying fixed stenotic lesion predisposes to in-situ thrombosis (36). The mechanisms driving EAR extend beyond simple re-thrombosis at a site of endothelial denudation. Mechanical manipulation during thrombectomy can provoke intimal dissection with exposure of subendothelial collagen, triggering the extrinsic coagulation cascade and platelet aggregation. In ICAS-related occlusions, disruption of an unstable atherosclerotic plaque releases a potent thrombogenic substrate. Residual thrombus fragments—too small to be retrieved by stent-retriever or aspiration devices but large enough to serve as nidi for propagating thrombosis—may remain adherent to the vessel wall. Additionally, post-procedural hypoperfusion states, whether due to impaired cardiac output, systemic hypotension during general anesthesia, or downstream microvascular resistance, reduce flow velocity and create conditions favoring stasis-related coagulation.

Pharmacological strategies to mitigate EAR have centered on glycoprotein (GP) IIb/IIIa receptor inhibitors, particularly tirofiban, which blocks the final common pathway of platelet aggregation. These agents are more widely used in Asian stroke populations, where ICAS-related occlusions are more prevalent. Retrospective studies and small prospective series have suggested that intra-arterial or intravenous tirofiban administration during or immediately after thrombectomy may reduce reocclusion rates and improve functional outcomes (45). Randomized controlled trial data, however, have yielded inconsistent results, reflecting the etiological heterogeneity of stroke: patients with ICAS-related occlusions appear to derive the greatest benefit, whereas those with cardioembolic stroke may face an unfavorable risk–benefit profile owing to elevated hemorrhagic risk without meaningful reduction in reocclusion (46). Procedural strategies—including the use of balloon guide catheters to arrest antegrade flow and prevent distal embolization during retrieval, direct aspiration as a primary or adjunctive technique, and minimization of the number of device passes through the occluded segment—are considered beneficial on pathophysiological grounds and are supported by observational data, though prospective randomized evidence confirming their impact on EAR specifically remains lacking (35).

### 3.4 Collateral circulation dynamics

The cerebral collateral circulation—comprising the circle of Willis, leptomeningeal anastomoses, and external carotid–internal carotid anastomotic pathways—serves as a dynamic, physiological buffer that sustains perfusion to the ischemic penumbra during arterial occlusion (47). Robust collateral flow slows the rate of infarct core expansion, preserves potentially viable tissue, and effectively extends the therapeutic window for recanalization. Patients with good collateral supply to the affected territory are more likely to present with a favorable “small core, large penumbra” profile—the very tissue pattern that defines candidacy for late-window thrombectomy in trials such as DAWN and DEFUSE 3 (6, 7).

Collateral function, however, is not static. Over the course of ischemia, initially adequate collateral pathways may fail progressively—a process driven by exhaustion of vasodilatory reserve in the arteriolar bed, increasing downstream resistance from edema and microvascular obstruction, and systemic hemodynamic fluctuations (48). This temporal dimension of collateral failure is poorly captured by current assessment tools. Single-phase CTA, the most commonly used modality, acquires images at a single time point and evaluates collateral extent using semiquantitative grading systems. The ASITN/SIR scale grades collateral filling from 0 (no collaterals visible) through 4 (complete collateral filling of the occluded territory with rapid retrograde opacification), while the Tan score employs a simpler three-tier system (0 = absent, 1 = filling ≤50% of the occluded territory, 2 = filling >50%) (49, 50). Although these scales provide useful prognostic stratification, their interrater agreement is only moderate (*κ* ≈ 0.4–0.6), and a single-phase acquisition cannot distinguish between collaterals that fill rapidly—suggesting adequate driving pressure—and those that fill slowly, which may indicate marginal hemodynamic reserve. Multiphase CTA addresses this limitation by acquiring three temporally staggered acquisitions (peak arterial, mid-venous, and late venous phases), enabling classification of collaterals as fast-filling versus slow-filling and achieving improved interrater consistency (*κ* ≈ 0.7–0.8) (27). Four-dimensional CT angiography (4D-CTA) provides even finer temporal resolution but demands greater computational resources and radiation exposure, and standardized interpretation criteria have not been established (28). Perhaps the most fundamental unresolved question is whether collateral adequacy is causally related to FR or whether it merely serves as a surrogate marker for the rate of tissue progression—a distinction with important implications for whether collateral augmentation strategies could directly reduce FR incidence (29).

### 3.5 Reperfusion injury and hemorrhagic transformation

Reperfusion of ischemic tissue, while essential for neuronal salvage, paradoxically initiates a secondary cascade of injury. The sudden restoration of oxygenated blood to tissue that has undergone a period of anaerobic metabolism generates a burst of reactive oxygen species (superoxide anion, hydroxyl radicals, peroxynitrite) through mitochondrial electron transport chain dysfunction and activation of xanthine oxidase and NADPH oxidase (51). These free radicals overwhelm endogenous antioxidant defenses and damage lipid membranes, proteins, and nucleic acids. In parallel, reperfusion activates both innate and adaptive inflammatory pathways: infiltrating neutrophils release proteases and additional oxidants, while microglia transition to a pro-inflammatory phenotype, producing cytokines (TNF-*α*, IL-1β, IL-6) that amplify tissue destruction. The blood–brain barrier (BBB), already compromised by ischemia-induced endothelial tight junction degradation, undergoes further disruption through matrix metalloproteinase (MMP-2, MMP-9)–mediated basement membrane proteolysis and rapid endothelial cytoskeletal reorganization, enabling extravasation of plasma proteins, inflammatory cells, and ultimately erythrocytes (44).

The most clinically consequential manifestation of reperfusion injury is hemorrhagic transformation (HT), which occurs in 10–40% of patients following thrombectomy depending on the definition employed. The European Cooperative Acute Stroke Study (ECASS) classification distinguishes hemorrhagic infarction (HI) from parenchymal hematoma (PH): HI type 1 denotes small petechiae along the infarct margins, HI type 2 refers to more confluent petechiae within the infarcted area without space-occupying effect, PH type 1 indicates a hematoma occupying ≤30% of the infarct zone with mild mass effect, and PH type 2 describes a dense hematoma exceeding 30% of the infarct with significant mass effect or hemorrhage remote from the infarct (52). Symptomatic intracranial hemorrhage (sICH)—variably defined across studies but generally requiring neurological deterioration attributable to hemorrhage—is the most feared complication and substantially elevates FR risk, with an odds ratio of approximately 7 (52, 53). Prediction models for HT (HAT score, SEDAN score, SITS-SICH model) incorporate variables such as baseline glucose, NIHSS score, early CT hypodensity, and time to treatment, but their discriminative performance remains modest, limiting their utility for individualized decision-making at the bedside (53). Imaging markers of BBB integrity—including post-contrast parenchymal enhancement on CT and permeability maps derived from perfusion imaging—may provide complementary predictive information, though their integration into clinical protocols requires further validation (54).

The relationship between time-to-reperfusion and the severity of reperfusion injury is not linear. The DAWN and DEFUSE 3 trials demonstrated that patients with favorable tissue profiles can benefit from thrombectomy at 16–24 h—a finding that would be paradoxical if reperfusion injury were simply a function of ischemic duration (6, 7). Rather, the extent of reperfusion injury appears to depend on the volume of irreversibly injured tissue at the time of recanalization, the degree of pre-existing BBB compromise, and individual variation in inflammatory and oxidative stress responses. This observation reinforces the primacy of tissue status over absolute time in governing the net therapeutic effect of recanalization.

### 3.6 Mechanistic interactions and the pathological cascade

These pathological processes are tightly coupled: each potentiates the others, forming a self-reinforcing network in which distal embolization serves as a critical link. Fragmentation of thrombus during device manipulation—or spontaneous downstream migration of clot debris—can occlude distal branches and arterioles that are not fully captured by conventional angiographic grading, producing an apparently successful mTICI 2b–3 result while leaving patchy areas of persistent hypoperfusion. This incomplete tissue-level reperfusion interacts bidirectionally with microvascular no-reflow: distal branch obstruction reduces capillary inflow, whereas endothelial swelling, leukocyte plugging, and microthrombus formation further impair downstream flow and promote secondary stasis (36, 39). In parallel, no-reflow alters local hemodynamics in ways that favor in-situ thrombosis and increase the risk of early arterial reocclusion; conversely, reocclusion intensifies downstream hypoperfusion and exacerbates capillary-level obstruction. Collateral circulation modulates the severity of reperfusion injury along comparable lines: robust collaterals may delay irreversible tissue damage during the ischemic phase, yet can also expose a larger volume of partially injured tissue to oxidative and inflammatory injury when flow is abruptly restored (48, 51). All of these processes converge on inflammatory amplification, perpetuating progressive tissue loss even after technically successful recanalization (43).

Several limitations constrain current mechanistic understanding. Animal models of stroke—typically employing filament-based middle cerebral artery occlusion in young, healthy rodents—fail to reproduce the complexity of human cerebrovascular disease, including chronic collateral adaptation, atherosclerotic vascular pathology, and comorbidity-related endothelial dysfunction. Clinical studies are predominantly cross-sectional, capturing pathological processes at single time points rather than tracking their dynamic evolution. Tools capable of quantifying the simultaneous activity of multiple FR mechanisms in real time within individual patients do not yet exist. Advancing toward mechanism-based, individualized therapeutic strategies will require the development of high-resolution, multimodal monitoring technologies; the construction of computational models that integrate clinical, imaging, and molecular data to characterize the dominant pathological process in each patient; and the systematic evaluation of interventions targeted to specific mechanistic subtypes of FR.

## 4 The tissue clock concept: redefining the temporal dimension of stroke treatment

### 4.1 Theoretical foundation

The axiom “time is brain” has guided acute stroke treatment for over two decades, underpinned by estimates that approximately 1.9 million neurons are lost per minute of untreated large vessel occlusion (11). The implicit assumption is that ischemic injury progresses at a roughly uniform rate across all patients, justifying rigid time-based treatment windows. Imaging evidence has comprehensively refuted this assumption. Serial diffusion-weighted MRI and perfusion studies demonstrate that the rate of infarct core expansion varies by an order of magnitude between individuals: some patients exhibit core growth rates exceeding 10 mL per hour, while others show virtually no measurable expansion over many hours (12, 55). This variability is governed by a constellation of factors—including the robustness and recruitment capacity of the leptomeningeal collateral circulation, the presence and severity of proximal and distal vascular pathology, systemic hemodynamic status, and the metabolic reserve of the affected tissue.

The tissue clock concept was proposed to capture this heterogeneity, reframing treatment eligibility from “how much time has elapsed since symptom onset?” to “how much salvageable tissue remains at the moment of clinical decision-making?” (12, 13). Stroke is not a single disease entity but a heterogeneous syndrome encompassing a spectrum of progression rates. At one extreme, “fast progressors”—typically characterized by absent or poor collateral circulation (ASITN/SIR grade 0–1), large vessel occlusions with limited distal reconstitution, or high metabolic demand in the affected territory—may complete the transition from penumbra to irreversible infarct within the first one to two hours. At the other extreme, “slow progressors” with extensive leptomeningeal collateral networks (ASITN/SIR grade 3–4, or Tan grade 2 on single-phase CTA) may sustain a viable penumbra for 12 h or longer. This biological reality mandates a paradigm shift from uniform time thresholds to tissue-based, individualized patient selection.

### 4.2 Validation through landmark trials: DAWN and DEFUSE 3

The DAWN and DEFUSE 3 trials provided definitive evidence that imaging-guided patient selection enables safe and effective mechanical thrombectomy well beyond the conventional 6-h window, validating the tissue clock concept in clinical practice (6, 7).

The DAWN trial (Clinical Mismatch in the Triage of Wake Up and Late Presenting Strokes Undergoing Neurointervention with Trevo) enrolled patients presenting 6 to 24 h after last known well, employing clinical-core mismatch criteria stratified by age. For patients aged 80 years or older, eligibility required an infarct core volume of less than 21 mL (assessed by diffusion-weighted MRI or CT perfusion using RAPID software) with an NIHSS score of 10 or greater. For patients younger than 80 years, two tiers were defined: core volume less than 31 mL with NIHSS ≥10, or core volume 31–51 mL with NIHSS ≥20. The trial demonstrated a striking benefit, with the adjusted difference in mean 90-day mRS utility-weighted score favoring thrombectomy (adjusted difference 2.0 points; 95% CI: 1.1–2.8), and the rate of functional independence (mRS 0–2) was 49% in the thrombectomy group versus 13% with medical management alone.

The DEFUSE 3 trial (Endovascular Therapy Following Imaging Evaluation for Ischemic Stroke) enrolled patients in the 6- to 16-h window using a perfusion imaging-based target mismatch profile. Eligibility required an ischemic core volume of less than 70 mL, a mismatch ratio of 1.8 or greater (defined as the ratio of critically hypoperfused tissue [Tmax >6 s] to ischemic core [cerebral blood flow <30% of normal]), and an absolute mismatch volume of at least 15 mL. The trial was stopped early for efficacy: the rate of functional independence at 90 days was 45% with thrombectomy versus 17% with medical therapy, and the treatment effect was consistent across pre-specified subgroups.

These results transformed clinical practice, but important caveats must be considered. The strict imaging selection criteria meant that only 10–15% of patients presenting in the extended time window met eligibility for enrollment (18). Single-center screening data suggest that approximately 38% of extended-window patients undergo advanced imaging evaluation, and of these, only a fraction satisfy the mismatch criteria (56). The high selectivity of these trials may therefore overestimate the magnitude of benefit that would be observed in broader clinical application. Moreover, the trials demonstrated efficacy in a carefully monitored research setting; whether equivalent outcomes are achievable in routine practice—where imaging interpretation, workflow efficiency, and procedural expertise may vary—remains an important question for implementation science.

Recent observational studies have explored thrombectomy beyond the 24-h window in highly selected patients with favorable tissue profiles, with some reporting preserved treatment benefit (57). While limited by observational design and small sample sizes, these data further reinforce the primacy of tissue status over arbitrary time boundaries and suggest that the therapeutic window may extend even further in appropriately selected individuals.

### 4.3 Imaging biomarkers for tissue clock quantification

Several imaging biomarkers have been investigated as quantitative surrogates for tissue clock status, each capturing a different dimension of the ischemic process.

The DWI-FLAIR mismatch pattern—defined as the presence of a diffusion-positive lesion in the absence of a corresponding signal change on fluid-attenuated inversion recovery (FLAIR) MRI—exploits the temporal lag between cytotoxic edema (detectable immediately on DWI) and vasogenic edema (which produces FLAIR signal change typically 3–6 h after onset). The WAKE-UP trial demonstrated that this imaging signature can identify patients within the thrombolytic treatment window even when symptom onset time is unknown, enabling thrombolysis in wake-up stroke with improved functional outcomes (58, 59). As a tissue clock indicator, the DWI-FLAIR mismatch provides a binary “early versus established” classification rather than a continuous measure of tissue viability. Its sensitivity is limited by substantial inter-individual variability in the rate of FLAIR signal evolution, which is influenced by the degree of collateral perfusion, lesion location and volume, and MRI acquisition parameters including field strength, inversion time, and echo time (13).

Net water uptake (NWU) quantifies the degree of cytotoxic edema by measuring the density difference between the ischemic territory and the homologous contralateral region on noncontrast CT, expressed as a percentage. The physiological basis is straightforward: as ischemic neurons lose the ability to maintain ionic homeostasis, intracellular water accumulation reduces tissue density in a manner proportional to the severity and duration of ischemic insult. Studies have established that NWU values exceeding 10–12% are associated with severe, likely irreversible edema and predict poor functional outcomes independent of other imaging markers (60, 61). Importantly, among patients with comparable ASPECTS scores and similar time from onset, elevated NWU identifies a subgroup with more advanced tissue injury and higher FR risk, suggesting that NWU captures pathophysiological information beyond what topographic scoring alone provides (62). Practical barriers to widespread adoption include the requirement for dedicated software with standardized region-of-interest placement protocols, the lack of large-scale threshold validation studies, and interference from bilateral ischemic lesions or pre-existing encephalomalacia that precludes reliable contralateral comparison.

Collateral circulation assessment, as discussed in detail in section 2.4, determines what has been termed the “burning rate” of the penumbra—the speed at which potentially salvageable tissue progresses to irreversible infarction (47, 63). Patients with robust collateral filling (ASITN/SIR grade 3–4, multiphase CTA score ≥4 out of 6) exhibit slow infarct growth and are disproportionately represented among late-window treatment responders. The hypoperfusion intensity ratio (HIR), calculated as the ratio of severely hypoperfused tissue volume (Tmax >10 s) to total perfusion lesion volume (Tmax >6 s), provides a perfusion-based surrogate for collateral adequacy: HIR values exceeding 0.4 indicate that a large proportion of the perfusion lesion is severely hypoperfused, consistent with poor collateral reserve and rapid tissue progression (64). While HIR correlates with angiographic collateral grading and infarct growth rate, it remains an indirect hemodynamic measure susceptible to confounding by systemic blood pressure, cardiac output, and intravascular volume status. Moreover, in patients with large established infarct cores, the predictive value of collateral assessment diminishes because the tissue damage has already progressed beyond the point where collateral adequacy meaningfully influences outcome.

### 4.4 Challenges in clinical translation

Despite its conceptual appeal and trial-level validation, the tissue clock framework faces substantial obstacles to broad clinical implementation. First, there is no unified quantification standard: the imaging thresholds employed in DAWN (core volume cutoffs stratified by age) differ fundamentally from those in DEFUSE 3 (perfusion-based mismatch criteria), and the relationship between these and alternative tissue clock markers such as NWU and DWI-FLAIR mismatch has not been formally integrated into a single decision framework (65). Second, the biological determinants of individual variation in tissue progression rate—the very factors that make the tissue clock concept necessary—remain incompletely characterized. Third, current imaging assessments provide a single cross-sectional “snapshot” of tissue status; the optimal timing for this assessment relative to the treatment decision, and the extent to which tissue status may change between imaging and recanalization, are not well established.

The imaging infrastructure required for tissue-based selection also raises concerns about equity of access. Perfusion CT or MR imaging, automated processing software, and expert interpretation are concentrated in comprehensive stroke centers, predominantly in urban areas of high-income countries. Primary stroke centers lacking these capabilities must transfer patients to higher-level facilities, a process that introduces delays potentially negating the benefit of tissue-based selection. Streamlined imaging protocols, telemedicine-supported remote interpretation, and development of simpler tissue clock surrogates (such as NWU derived from widely available noncontrast CT) represent potential strategies to mitigate this disparity. At the systems level, the additional complexity of tissue-based decision-making—requiring acquisition, processing, and interpretation of advanced imaging before treatment authorization—may paradoxically increase door-to-puncture times if not embedded within carefully designed workflow protocols (34, 66).

### 4.5 Future directions

Artificial intelligence holds considerable promise for advancing tissue clock assessment. Machine learning algorithms trained on multimodal imaging datasets may integrate spatial perfusion patterns, collateral morphology, core–penumbra dynamics, and clinical variables into a composite tissue viability estimate that exceeds the predictive capacity of any single parameter (67, 68). A critical barrier, however, is the “black box” nature of deep learning models: clinicians are understandably reluctant to base irreversible treatment decisions on algorithms whose reasoning cannot be interrogated. Explainable AI approaches—including attention maps that highlight the image regions driving a prediction, and model-agnostic interpretability frameworks—may bridge this gap, but their clinical utility requires prospective evaluation.

The integration of circulating biomarkers with imaging represents another frontier. Glial fibrillary acidic protein (GFAP), released upon astrocytic injury, and neurofilament light chain (NfL), a marker of axonal damage, can be measured in peripheral blood within hours of stroke onset and may provide complementary information about the severity of ongoing tissue destruction before imaging is available or when imaging is equivocal. Point-of-care devices enabling rapid bedside measurement of these analytes could support pre-hospital triage decisions, particularly in settings where advanced imaging is not immediately accessible.

Dynamic monitoring technologies—including continuous transcranial Doppler sonography for real-time assessment of cerebral blood flow velocity, and near-infrared spectroscopy for noninvasive estimation of regional cortical oxygenation—offer the potential to track tissue status longitudinally rather than relying on single-timepoint assessments. Current accuracy limitations preclude standalone clinical use, but these modalities may prove valuable as adjuncts to conventional imaging within multimodal monitoring protocols.

The tissue clock concept has advanced stroke treatment from a uniform, time-governed paradigm toward individualized, pathophysiology-driven decision-making. Realizing its full potential will require convergence across several domains: standardized quantification frameworks that integrate multiple tissue clock indicators, prospective validation of AI-assisted decision tools in diverse clinical settings, and equitable access strategies that extend precision stroke care beyond specialized academic centers. The ultimate question that the tissue clock seeks to answer is not “how long since symptom onset?” but “how much salvageable tissue remains, and can we recover it?”.

## 5 Assessment and application of imaging biomarkers

### 5.1 Noncontrast CT and ASPECTS

Noncontrast CT (NCCT) remains the first-line imaging modality in acute stroke evaluation, offering rapid acquisition, near-universal availability, and reliable exclusion of intracranial hemorrhage. Early ischemic changes on NCCT—including sulcal effacement, loss of grey-white matter differentiation, and parenchymal hypodensity—provide the substrate for the Alberta Stroke Program Early CT Score (ASPECTS), a 10-point semiquantitative scale in which one point is subtracted for each of 10 predefined regions within the middle cerebral artery (MCA) territory showing evidence of early infarction (23, 69). A score of 10 indicates no visible early ischemic changes, while lower scores reflect more extensive territorial involvement. The relationship between ASPECTS and FR is well established: patients with ASPECTS ≤5 exhibit markedly reduced rates of functional independence, and each one-point decrement is associated with an approximately 20% increase in FR risk (70).

Several limitations, however, constrain the clinical utility of ASPECTS as a standalone decision tool. Interrater reliability is moderate at best (*κ* ≈ 0.6–0.7 among neuroradiologists) and falls substantially among less experienced readers, including emergency physicians who frequently perform initial assessments (24). Scan acquisition parameters—including kilovoltage, slice thickness, and the window width and level settings used for interpretation—can meaningfully alter the visibility of subtle early ischemic changes, introducing a source of variability that is independent of the reader. From a neuroanatomical standpoint, the binary, region-based scoring approach treats all territories as equivalent despite marked differences in their functional significance: a one-point deduction for involvement of the caudate nucleus or internal capsule—deep structures whose injury commonly produces severe, persistent motor deficits—carries the same numerical weight as involvement of a cortical insular ribbon region, an asymmetry that may compromise prognostic accuracy.

Automated ASPECTS tools, most notably RAPID ASPECTS and e-ASPECTS, have been developed to reduce subjectivity and enable faster clinical decision-making. These platforms achieve agreement with expert neuroradiologists in the range of 0.7–0.8, suggesting performance comparable to but not yet exceeding human assessment (25, 26). Their principal advantages lie in consistency, speed, and the potential for deployment in settings lacking subspecialty neuroradiology coverage. Current limitations include strong dependence on image quality and acquisition protocol, limited generalizability across CT scanner manufacturers and software versions, and the absence of established protocols for managing discrepancies between automated and clinician-derived scores.

### 5.2 Net water uptake

Net water uptake (NWU) extends the information obtainable from NCCT by providing a quantitative, continuous measure of cytotoxic edema severity rather than a categorical topographic assessment. The calculation compares Hounsfield unit (HU) density within the ischemic territory to the homologous contralateral region: NWU (%) = (1 − density_ischemic/density_normal) × 100. As ischemic cells lose ionic homeostasis and accumulate intracellular water, tissue density decreases in proportion to the severity and chronicity of injury. Studies have established that NWU exceeding 10–12% identifies tissue with severe, likely irreversible edema and predicts poor functional outcomes (60, 61). A key finding is that NWU provides prognostic information independent of ASPECTS: among patients with identical ASPECTS scores and similar times from symptom onset, those with elevated NWU demonstrate significantly higher FR rates, indicating that NWU captures a dimension of tissue injury—specifically, the degree of cellular bioenergetic failure—that topographic scoring alone misses (62).

The concept of NWU as a tissue clock marker is therefore compelling: it quantifies how far along the ischemic cascade a given region of tissue has progressed, regardless of the elapsed clock time. Practical implementation, however, faces barriers including the requirement for dedicated software with standardized and reproducible region-of-interest placement, the absence of validated thresholds from large prospective cohorts, and interference in patients with bilateral ischemic lesions, prior infarcts, or significant leukoaraiosis where contralateral reference tissue may not be truly normal. Whether NWU will transition from a promising research tool to a routine clinical biomarker will depend on the development of automated, scanner-agnostic quantification pipelines and their integration into existing imaging workflows.

### 5.3 CT and MR perfusion imaging

Perfusion imaging—whether CT perfusion (CTP) or MR perfusion (using dynamic susceptibility contrast or arterial spin labeling)—provides the most direct *in vivo* assessment of tissue hemodynamic status and forms the imaging backbone of tissue clock-based patient selection. The ischemic core is most commonly delineated as tissue with cerebral blood flow (CBF) less than 30% of the mean normal value, while the perfusion lesion (representing the combined core and penumbra) is defined by a time-to-maximum (Tmax) exceeding 6 s; the mismatch between perfusion lesion and core volumes estimates the volume of tissue that is hemodynamically compromised but potentially salvageable (30, 31, 71). These thresholds, validated in the DEFUSE and DEFUSE 2 cohorts and subsequently operationalized in the DAWN and DEFUSE 3 trials, have become the de facto standard for extended-window patient selection (6, 7).

The evidence supporting perfusion-guided selection is robust for the specific populations enrolled in these trials, but several important caveats apply. First, the trials were highly selective: single-center screening data indicate that only approximately 38% of extended-window patients undergo perfusion imaging evaluation, and of those evaluated, only 10–15% satisfy the mismatch criteria for treatment (18, 56). The magnitude of benefit observed in trials may therefore not be fully generalizable to broader clinical populations. Second, the CBF 30% and Tmax 6-s thresholds are empirical values derived from specific study cohorts; they have not been validated across the full range of stroke etiologies (cardioembolic versus atherosclerotic versus dissection-related), patient demographics, and hemodynamic states. For instance, patients with chronic carotid stenosis and longstanding hemodynamic compensation may exhibit baseline perfusion asymmetries that confound threshold-based classification. Third, substantial inter-software variability exists: comparative studies have documented 20–40% differences in core and penumbra volume estimates across commercial perfusion platforms, with inter-software agreement coefficients of only 0.7–0.8, raising concerns about the reproducibility of treatment decisions that hinge on absolute volume thresholds (32). Finally, a single perfusion acquisition provides a cross-sectional hemodynamic snapshot whose appearance is influenced by the time elapsed since onset, the dynamic state of collateral circulation, and systemic hemodynamic variables—factors that may change between imaging and treatment.

### 5.4 Hypoperfusion intensity ratio as a collateral surrogate

The hypoperfusion intensity ratio (HIR) offers a perfusion-derived, quantitative surrogate for collateral circulatory adequacy. Calculated as the ratio of severely hypoperfused tissue volume (Tmax >10 s) to the total perfusion lesion volume (Tmax >6 s), HIR reflects the proportion of the ischemic territory that has progressed from moderate to severe hemodynamic compromise (64). A higher HIR value indicates that a larger fraction of the perfusion lesion is experiencing critically low perfusion, consistent with inadequate collateral supply and rapid tissue progression. HIR values exceeding 0.4 have been associated with poor collateral grading on CTA, accelerated infarct growth on follow-up imaging, and unfavorable clinical outcomes. The principal advantage of HIR is that it is derived from the same perfusion dataset already acquired for core–penumbra assessment, requiring no additional imaging sequences or contrast administration. Its limitations include susceptibility to confounding by systemic hemodynamic variables—blood pressure, cardiac output, and intravascular volume all influence perfusion transit times independently of collateral status—and diminished predictive utility in patients with large established infarct cores, where the prognosis is already poor regardless of residual collateral function.

### 5.5 Advanced MRI biomarkers

Several MRI-derived markers provide complementary information for tissue clock assessment, each with distinct strengths and limitations.

The DWI-FLAIR mismatch pattern, as detailed in section 3.3, identifies patients likely to be within 4.5 h of symptom onset by exploiting the temporal dissociation between cytotoxic edema (DWI-positive) and vasogenic edema (FLAIR-positive). The WAKE-UP trial validated its use for selecting wake-up stroke patients for intravenous thrombolysis (58, 59). Its sensitivity, however, is reduced for small lesions and posterior circulation infarcts, and individual variation in the rate of FLAIR signal evolution—driven by differences in collateral perfusion, lesion volume, and scanning parameters (field strength, inversion time, repetition time)—introduces uncertainty into the temporal estimate.

FLAIR vascular hyperintensity (FVH), characterized by serpiginous high signal within cortical sulci on FLAIR sequences, is thought to reflect slow retrograde flow through leptomeningeal collateral channels. Its presence has been variably associated with both favorable outcomes (interpreted as evidence of collateral supply maintaining tissue viability) and unfavorable outcomes (interpreted as indicating severe proximal hypoperfusion) across different studies. This contradictory literature likely reflects differences in the timing of imaging relative to onset, the criteria used to define FVH, and the heterogeneity of the underlying collateral physiology. In the absence of standardized grading criteria and prospective validation, FVH remains an investigational marker.

Susceptibility-weighted imaging (SWI) detects cerebral microbleeds (CMBs) as small, hypointense foci resulting from hemosiderin deposition in perivascular macrophages—a signature of prior microhemorrhage. The presence and distribution of CMBs provide indirect information about the integrity of the cerebral microvasculature: lobar CMBs suggest cerebral amyloid angiopathy (CAA), while deep or mixed distributions point toward hypertensive vasculopathy. Multiple CMBs (typically defined as ≥5) have been associated with a 3- to 5-fold increase in the risk of symptomatic intracranial hemorrhage following reperfusion therapy, supporting their use in perioperative hemorrhagic risk stratification (72). The direct association between CMB burden and FR risk, however, has not been firmly established, and the incremental predictive value of SWI above standard imaging parameters warrants further investigation.

### 5.6 Vascular imaging and collateral assessment

Comprehensive assessment of the cervicocranial vasculature by CT angiography (CTA) serves dual purposes: confirming the site and extent of large vessel occlusion and evaluating the status of collateral circulation. Multiple collateral grading systems have been proposed, each with trade-offs between simplicity and granularity. The Tan score classifies collateral filling of the MCA territory into three tiers (0 = absent, 1 = filling ≤50% of the occluded territory, 2 = filling >50%), offering simplicity at the cost of reduced discrimination. The ASITN/SIR scale provides a more granular five-tier classification: grade 0 indicates no visible collaterals to the ischemic territory, grade 1 slow collateral filling to the periphery of the ischemic bed with persistence of some defect, grade 2 rapid collateral filling to the periphery with persistence of defect and delayed filling of the involved territory, grade 3 slow but complete filling of the ischemic bed via collateral pathways by the late venous phase, and grade 4 rapid and complete collateral filling by retrograde perfusion. The regional leptomeningeal collateral (rLMC) score provides territory-specific evaluation, scoring collateral opacification across individual cortical regions (49, 50). Across these systems, interrater agreement is only moderate (*κ* ≈ 0.4–0.6), reflecting the subjective nature of visual collateral assessment on single-phase imaging.

Multiphase CTA (mCTA) addresses the fundamental temporal limitation of single-phase acquisitions by imaging at three time points—peak arterial, mid-venous, and late venous phases—enabling differentiation of collateral pathways that fill rapidly (suggesting robust driving pressure) from those that fill slowly (suggesting marginal reserve). The multiphase CTA collateral grading system employs a 6-point scale (0–5) that integrates the extent and timing of collateral opacification, achieving substantially improved interrater consistency (*κ* ≈ 0.7–0.8) compared with single-phase methods (27). The trade-offs are increased radiation exposure (approximately 2–3 additional low-dose acquisitions), greater contrast agent load, and increased reading complexity.

Quantitative approaches to collateral assessment—including automated measurement of collateral territory opacification volumes, calculation of collateral indices (ratio of ipsilateral to contralateral vessel opacification), and analysis of time-density curves from 4D-CTA—represent methodological advances toward more objective, reproducible evaluation (28). These techniques reduce subjectivity and offer continuous rather than ordinal measures of collateral function, but their computational demands and implementation complexity currently restrict adoption to research settings. Across all modalities, the fundamental clinical question—“how much collateral flow is sufficient to sustain tissue viability?”—remains without a universally accepted quantitative answer.

### 5.7 Emerging and complementary technologies

Several imaging technologies are under investigation as adjuncts or alternatives to established modalities.

Arterial spin labeling (ASL) MRI provides contrast-free perfusion assessment by magnetically labeling arterial blood water as an endogenous tracer, eliminating the need for gadolinium administration and enabling repeated measurements without cumulative contrast exposure (73). In the post-recanalization setting, ASL can identify regions of persistent hypoperfusion suggestive of microvascular no-reflow, as well as areas of luxury perfusion that may indicate loss of autoregulatory capacity. Its principal limitations are lower signal-to-noise ratio compared with contrast-based methods, longer acquisition times (typically 3–5 min), and sensitivity to motion artifact and transit-time delays, all of which constrain its utility in the hyperacute clinical setting.

Emerging evidence suggests that impaired cerebral venous outflow—assessed by the prominence and timing of venous opacification on CTA or MR venography—may independently correlate with FR, potentially by exacerbating tissue edema and impairing microcirculatory drainage even when arterial inflow is successfully restored (74). This concept remains at an early stage of investigation, with standardized quantification methods and validation in prospective cohorts still required.

Dual-energy CT (DECT) exploits the differential attenuation of iodine contrast and blood products at two different energy levels to distinguish post-procedural contrast extravasation (benign, often related to BBB disruption) from true parenchymal hemorrhage (75). This distinction is clinically important because it directly influences decisions about early antithrombotic therapy: contrast staining is a transient, self-limited phenomenon that does not contraindicate anticoagulation or antiplatelet therapy, whereas hemorrhage requires withholding such agents. The availability of DECT remains limited to facilities equipped with dual-source or rapid kV-switching CT scanners, and performance may be compromised by patient motion artifact during acquisition.

### 5.8 Multimodal integration and the path toward standardization

The accumulated evidence makes clear that no single imaging biomarker adequately captures the complexity of tissue status in acute ischemic stroke. Composite assessment strategies that integrate complementary parameters—such as ASPECTS for territorial extent, perfusion core and penumbra volumes for hemodynamic compromise, collateral grading for tissue progression rate, and NWU for edema severity—consistently outperform any individual metric in predicting both FR risk and functional outcome (19, 76). Clinical-imaging composite scores, including the SELECT score (which incorporates age, NIHSS, and imaging variables), represent early efforts at such integration and have demonstrated promising discriminative performance across multiple validation cohorts.

The path from multimodal integration in research to standardized clinical implementation, however, faces formidable obstacles. The optimal weighting of different parameters within combined models has not been determined, and it is unclear whether a single weighting scheme can apply across the diversity of stroke subtypes, etiologies, and patient populations. Machine learning approaches offer a natural framework for learning these weights from data, but they inherit the challenges of interpretability, overfitting risk in small datasets, and uncertain generalizability across institutions with different imaging equipment, software platforms, and patient demographics. Cross-device and cross-software variability in fundamental measurements—core volume, perfusion thresholds, collateral grading—creates a heterogeneity that undermines the reproducibility of any algorithm trained on data from a single platform. International consensus efforts, including the Stroke Imaging Research (STIR) consortium and the Stroke Neuro-Imaging Phenotype (SONIIA) initiative, have articulated standardization goals but have encountered inconsistent implementation across participating centers (65). Achieving true standardization will require coordinated engagement among device manufacturers, software developers, and clinical societies to establish harmonized acquisition protocols, post-processing algorithms, and reporting standards.

## 6 Prediction models, intervention strategies, and future directions

### 6.1 Prediction models: from clinical scores to computational algorithms

The evolution of FR prediction has progressed through three overlapping phases, each incorporating increasingly complex data and analytic methods.

Traditional clinical scoring systems represent the earliest approach. The Totaled Health Risks in Vascular Events (THRIVE) score combines age, baseline NIHSS, and the presence of comorbidities (hypertension, diabetes, atrial fibrillation) into a composite index that provides crude stratification of functional outcomes after endovascular treatment (77). The iScore, originally developed for prognostication after intravenous thrombolysis, has been adapted for thrombectomy populations but was not designed to incorporate imaging data central to tissue-based selection (78). The PRE score focuses specifically on FR prediction, incorporating three readily available variables—age greater than 75 years, ASPECTS of 6 or below, and baseline mRS of 2 or greater—to generate a simple risk estimate (19). While these scores offer practical bedside utility and require no specialized imaging, they share fundamental limitations: narrow variable dimensions that exclude imaging and procedural data, linear assumptions that fail to capture the complex, nonlinear interactions among predictors, and limited external validation across diverse populations and healthcare systems (38). Most importantly, they do not incorporate the tissue status information that forms the foundation of modern, imaging-guided treatment selection.

Machine learning (ML) methods have emerged as a second-generation approach, capable of integrating high-dimensional clinical and imaging data while modeling nonlinear predictor interactions. Gradient-boosted ensemble methods (such as XGBoost) trained on datasets combining clinical variables (age, NIHSS, comorbidities, time metrics) with imaging parameters (ASPECTS, core volume, collateral grade) have achieved pre-procedural prediction accuracy with area under the receiver operating characteristic curve (AUC) values of approximately 0.79; post-procedural models that additionally incorporate treatment-response variables—such as 24-h NIHSS, final recanalization grade, and number of device passes—reach AUC values as high as 0.91 (79). The Futile Recanalization Prediction Score (FRPS), developed using a large sample ML methodology and incorporating 12 clinical and imaging variables, stratifies patients into mild, moderate, and severe FR risk categories with promising initial discriminative performance (80). These advances notwithstanding, significant barriers impede clinical adoption. The “black box” nature of many ML algorithms limits clinician interpretability and trust—a critical concern when predictions influence irreversible treatment decisions. Many studies have been conducted on relatively small, single-center retrospective cohorts, raising concerns about overfitting and uncertain transferability to institutions with different patient demographics, imaging protocols, and treatment workflows. Furthermore, the inclusion of post-procedural variables in some models, while improving apparent predictive accuracy, substantially diminishes their utility for the pre-procedural decision point where risk stratification has the greatest impact on clinical management (67).

Imaging-driven models represent a third approach, employing deep learning architectures and radiomics to extract high-dimensional quantitative features directly from NCCT, CTP, or MRI source images, combined with clinical metadata, to predict FR (67, 81). These models have the theoretical advantage of capturing subtle imaging patterns—texture heterogeneity, boundary characteristics, perfusion spatial gradients—that are invisible to human readers. A critical and unresolved question, however, is whether the marginal gain in predictive accuracy relative to simpler models using readily available inputs (core volume, ASPECTS, collateral grade, age, NIHSS) justifies the substantial infrastructure costs of expert image annotation, rigorous quality control, and prospective multicenter validation. For pragmatic, broad-scale clinical implementation, simpler models with strong external validation may be preferable to complex algorithms with marginally superior performance in development cohorts.

### 6.2 Intervention strategies

Intervention strategies for reducing FR extend across the treatment chain, from patient selection through procedural technique to post-procedural critical care. Operationally, the tissue clock should inform not only the binary decision of whether to proceed with thrombectomy, but also how the procedure is performed and how post-reperfusion risk is managed. A patient with a favorable tissue profile may justify aggressive efforts to achieve first-pass near-complete reperfusion, whereas a patient with extensive established injury, strategic lesion location, or limited recovery reserve may require a more cautious appraisal of procedural burden and expected benefit. The specific strategy—or combination of strategies—should therefore be matched to the individual patient’s clinical, imaging, and biological risk profile, rather than applied uniformly across cases.

#### 6.2.1 Patient selection and shared decision-making

Extended-window imaging selection, as validated by DAWN and DEFUSE 3, has proven effective for identifying patients most likely to benefit from thrombectomy beyond the conventional time window. At the other end of the spectrum, patients with large established infarct cores (exceeding approximately 70–100 mL) have historically been excluded from treatment on the assumption that FR rates would be prohibitively high. Three recent landmark trials have challenged this assumption. The RESCUE-Japan LIMIT trial enrolled patients with ASPECTS 3–5 (corresponding to large core infarctions) and demonstrated that thrombectomy improved functional outcomes compared with medical management, with 31% achieving mRS 0–3 versus 12.7% in the control group (82). The SELECT 2 trial enrolled patients with core volumes of 50 mL or greater (or ASPECTS 3–5) within 24 h and showed a higher rate of functional independence with thrombectomy (20.0% mRS 0–2) versus medical therapy (6.9%) (83). The ANGEL-ASPECT trial, conducted primarily in Chinese centers, enrolled patients with ASPECTS 3–5 or core volume 70–100 mL and reported a functional independence rate of 30.0% with thrombectomy versus 11.6% with medical management (84). Across these trials, subgroup analyses consistently identified younger age, better residual collateral supply, and achievement of rapid, high-quality recanalization as the strongest predictors of benefit within the large-core population.

These findings have recalibrated—but not eliminated—the role of patient selection. For patients whose composite risk assessment indicates high FR probability but who face certain severe disability or death without intervention, treatment may still be appropriate after thorough discussion of realistic expectations with patients and families (85). The goal is not categorical denial of treatment but rather informed, shared decision-making that accounts for individual risk–benefit profiles, patient values, and the anticipated impact on quality of life.

#### 6.2.2 Procedural optimization: catheter technology and the first-pass effect

The technical execution of mechanical thrombectomy has emerged as a critical and modifiable determinant of clinical outcome. Among procedural variables, the first-pass effect (FPE)—defined as achieving near-complete or complete recanalization (mTICI 2c/3) on the initial device deployment—has been consistently associated with superior functional outcomes compared with recanalization requiring multiple passes (35). Each additional retrieval attempt is associated with incremental endothelial injury, increased risk of thrombus fragmentation with distal embolization into previously unaffected territories, prolonged procedural time, and greater contrast and radiation exposure. Observational data indicate that patients achieving FPE have approximately 15–20% higher rates of functional independence compared with those requiring two or more passes, even after adjusting for confounders such as clot burden, occlusion location, and collateral status.

Catheter technology is central to optimizing FPE rates and overall procedural success. The balloon guide catheter (BGC)—a large-bore guide catheter incorporating an inflatable proximal balloon that arrests antegrade flow in the internal carotid artery during device retrieval—represents one of the most impactful technical innovations in modern thrombectomy. By establishing flow arrest, the BGC creates a controlled hemodynamic environment that serves three purposes: preventing antegrade migration of thrombus fragments during retrieval, enabling reversal of flow direction to facilitate aspiration of debris, and reducing the risk of embolization into new vascular territories. Meta-analytic data comparing BGC-assisted thrombectomy with conventional guide catheter approaches have demonstrated significantly higher first-pass recanalization rates (approximately 56% versus 42%), lower rates of embolization to new territories (approximately 3% versus 9%), and improved functional outcomes (mRS 0–2 in approximately 59% versus 48%). The PROTECT PLUS registry, one of the largest observational datasets evaluating BGC use, confirmed these benefits in a real-world, multicenter setting. Despite this evidence, BGC utilization remains inconsistent across centers, partly because of the larger femoral sheath required (8–9 French), the additional procedural step of balloon inflation/deflation, and operator preference.

The choice of primary thrombectomy technique—stent retriever, direct aspiration, or a combined approach—represents another critical decision with implications for FPE and FR risk. Stent retrievers (such as Solitaire and Trevo) are deployed across the thrombus and withdrawn under flow arrest or aspiration, physically engaging and extracting the clot. Direct aspiration (the ADAPT technique) uses large-bore aspiration catheters (typically 0.060–0.072 inch inner diameter) advanced to the face of the thrombus, applying continuous suction to ingest the clot without stent deployment. The ASTER trial, which randomized patients to first-line contact aspiration versus first-line stent retriever, found no significant difference in the primary outcome of successful reperfusion (mTICI 2b/3) between techniques (85.4% versus 83.1%), though the aspiration group trended toward shorter procedure times. The COMPASS trial similarly found non-inferior recanalization rates with aspiration-first approaches. In current practice, many interventionists employ a combined or “Solumbra” technique—simultaneous stent retriever deployment with aspiration through a large-bore intermediate catheter—which may maximize clot engagement and retrieval efficiency, although randomized comparison against single-technique approaches remains limited. Device selection should be individualized based on clot location, vessel tortuosity, proximal access geometry, and operator experience.

#### 6.2.3 Hemodynamic management

Perioperative blood pressure management represents a modifiable factor with direct implications for both collateral maintenance during the procedure and reperfusion injury risk after recanalization. Systemic hypotension—whether from general anesthesia induction, sedation-related vasodilation, or inadequate fluid resuscitation—reduces the perfusion pressure gradient driving collateral flow, potentially accelerating penumbral tissue loss during the window between anesthetic induction and arterial recanalization (86). Intraoperative blood pressure monitoring with a target of maintaining systolic blood pressure above 140 mmHg (or mean arterial pressure above 70 mmHg) during the pre-recanalization phase is widely advocated, although prospective evidence establishing optimal intraoperative targets is lacking. Following successful recanalization, blood pressure management must balance the competing objectives of maintaining perfusion to newly revascularized—and potentially dysautoregulated—tissue against the risk of exacerbating hemorrhagic transformation. Observational data suggest that moderate systolic blood pressure (140–180 mmHg) in the first 12–24 h post-thrombectomy is associated with favorable outcomes, with both extremes (hypotension and sustained hypertension above 180 mmHg) increasing the risk of poor outcome (22). Patients with large infarcts, evidence of early BBB disruption, or contrast extravasation on post-procedure imaging may warrant more aggressive blood pressure reduction to minimize hemorrhagic conversion.

#### 6.2.4 Adjunctive pharmacotherapy

Pharmacological adjuncts to mechanical thrombectomy target the residual pathophysiological mechanisms that contribute to FR despite technically successful macrovascular recanalization.

Intra-arterial thrombolysis addresses microvascular residual thrombi that are beyond the reach of mechanical devices. The CHOICE trial demonstrated that low-dose intra-arterial alteplase (0.225 mg/kg, maximum 22.5 mg) administered directly into the recanalized territory immediately after thrombectomy improved the rate of near-complete or complete reperfusion (mTICI 2c/3) and trended toward better functional outcomes without a significant increase in symptomatic hemorrhage (87). The ongoing CHOICE-2 trial is designed to provide confirmatory evidence in a larger, multicenter cohort. This approach is pathophysiologically rational given that microvascular thrombosis is a principal driver of the no-reflow phenomenon; however, the optimal dose, timing, and patient selection criteria require further definition.

Immunomodulatory agents target the inflammatory component of reperfusion injury. Fingolimod, a sphingosine-1-phosphate receptor modulator that sequesters lymphocytes in secondary lymphoid organs and reduces their infiltration into ischemic tissue, demonstrated a reduction in infarct growth in a small randomized pilot study of acute ischemic stroke (88). Minocycline, a tetracycline-class antibiotic with pleiotropic anti-inflammatory and neuroprotective properties—including inhibition of microglial activation, suppression of MMP-9, and scavenging of reactive oxygen species—showed preliminary evidence of reduced hemorrhagic transformation risk in an open-label trial (89). Both agents require validation in adequately powered, controlled trials with prospective selection of patient populations most likely to benefit based on their inflammatory and tissue injury profiles.

Antiplatelet therapy for FR prevention must be guided by stroke etiology. In patients with ICAS-related occlusion—a population particularly susceptible to early reocclusion due to the underlying fixed stenotic lesion—GP IIb/IIIa inhibitors such as tirofiban may reduce reocclusion risk and improve outcomes, as suggested by retrospective analyses and small prospective series (45, 46). In contrast, patients with cardioembolic stroke mechanisms face increased hemorrhagic risk from potent antiplatelet therapy without a proportionate reduction in reocclusion, given that the primary re-thrombotic stimulus is cardiogenic embolism rather than local arterial disease. This etiological distinction underscores the importance of rapid, accurate stroke subtyping—ideally before or during the thrombectomy procedure—to guide adjunctive pharmacotherapy.

#### 6.2.5 Post-thrombectomy neurocritical care

The first 72 h following thrombectomy constitute a high-risk period during which secondary complications can convert a technically successful recanalization into a functionally futile outcome. Structured neurocritical care monitoring and management during this period is essential.

Malignant cerebral edema, peaking at 24–72 h after recanalization, represents the most immediately life-threatening complication. Early recognition through serial neurological assessment (at minimum every 1–2 h during the first 24 h, with vigilance for progressive obtundation, pupillary asymmetry, or contralateral motor deterioration) combined with scheduled follow-up imaging enables timely intervention. Medical management with hyperosmolar therapy (mannitol or hypertonic saline) provides temporary ICP reduction, while decompressive hemicraniectomy in appropriate candidates—particularly patients younger than 60 years with progressive deterioration despite maximal medical management—has been shown to reduce mortality, as demonstrated in the DECIMAL, DESTINY, and HAMLET trials (90).

Early reocclusion surveillance requires a high index of clinical suspicion. Acute neurological deterioration following initial post-procedural improvement should prompt urgent repeat vascular imaging—either CTA or point-of-care transcranial Doppler—to evaluate for reocclusion and enable consideration of secondary intervention (91). Transcranial Doppler monitoring, where available, can provide continuous hemodynamic surveillance of the recanalized vessel, though its sensitivity and specificity for detecting reocclusion have not been established in large prospective series.

Supportive care fundamentals—standardized glycemic control targeting normoglycemia while avoiding hypoglycemia, aggressive treatment of fever (targeting temperature <37.5 °C using pharmacological and surface cooling measures), oxygenation optimization to prevent secondary hypoxic injury, and protocolized blood pressure management—represent low-cost, high-yield interventions whose collective impact on functional outcomes is often underestimated. Seizure prophylaxis in high-risk patients (those with cortical involvement and hemorrhagic transformation) and rigorous infection prevention (aspiration pneumonia screening, early mobilization when safe, catheter minimization) further contribute to the prevention of secondary neurological deterioration (21).

### 6.3 Future directions

The convergence of technological innovation, expanding biological understanding, and evolving trial methodology opens several promising avenues for reducing FR incidence.

In the domain of artificial intelligence, the immediate priority is the development of real-time clinical decision support tools that integrate clinical, imaging, and laboratory data at critical decision nodes—whether to proceed with thrombectomy, when to terminate an unsuccessful retrieval attempt, and whether to add adjunctive pharmacotherapy. These tools must be interpretable, providing transparent reasoning that clinicians can evaluate and override, and must be embedded seamlessly within clinical workflows to avoid adding procedural friction or delay (67, 68). Beyond decision support, imaging automation is progressing from isolated ASPECTS scoring toward comprehensive “one-click” assessment platforms capable of simultaneously quantifying core volume, delineating the penumbra, grading collateral status, and estimating tissue viability—with the goal of reducing time-to-decision while maintaining or exceeding the accuracy of expert human interpretation.

Blood-based biomarkers hold promise as a complement to imaging. Neurofilament light chain (NfL), which reflects axonal injury with a temporal profile that evolves over hours to days, and glial fibrillary acidic protein (GFAP), released rapidly upon astrocytic damage, can be measured in peripheral blood and may provide early information about tissue destruction severity before imaging is completed or when imaging findings are equivocal. The development of point-of-care immunoassay devices for these analytes could support pre-hospital triage and intra-procedural decision-making, though clinical validation against imaging-defined tissue status is needed.

Combination treatment strategies addressing multiple FR mechanisms simultaneously—a “cocktail therapy” approach—may prove more effective than single-target interventions. For patients with high inflammatory biomarker profiles, an immunomodulatory-dominant regimen (fingolimod or minocycline combined with standard thrombectomy) may be appropriate; for those with ICAS-related occlusion and high reocclusion risk, an anti-platelet–dominant strategy (tirofiban plus optimized stenting) may be indicated; for patients with evidence of severe no-reflow, intra-arterial thrombolysis or collateral enhancement strategies may take priority. Adaptive platform trial designs, which efficiently evaluate multiple interventions within a single master protocol and enable arms to be added, dropped, or graduated based on pre-specified efficacy or futility criteria, offer a pragmatic framework for testing these mechanism-stratified combinations (57).

Standardization and quality governance remain essential prerequisites for advancing the field. Unified imaging acquisition, post-processing, and reporting frameworks are necessary for model generalization and multicenter research comparability (65). Registry databases—such as the Get With The Guidelines–Stroke (GWTG-Stroke) platform—provide the infrastructure for aggregating real-world evidence, benchmarking institutional performance, and identifying best practices across diverse clinical settings. Institutional quality dashboards that track key performance metrics—door-to-puncture time, first-pass effect rate, hemorrhagic complication rates, and functional outcome distributions—can drive continuous quality improvement and identify centers that may benefit from targeted educational interventions.

Finally, equitable access to precision stroke care must be addressed as a systemic priority. The imaging, computational, and procedural infrastructure required for tissue clock-guided treatment is concentrated in comprehensive stroke centers in high-income urban settings. Telemedicine stroke networks that connect community hospitals with expert centers for remote imaging interpretation and treatment decision support, streamlined imaging protocols adaptable to resource-limited settings, and investment in operator training across geographic regions are all necessary to prevent precision stroke care from becoming a benefit available only to privileged populations. The stroke community bears a responsibility to ensure that technological advances in FR prediction and prevention translate into improved outcomes for all patients, not merely those with access to the most advanced healthcare systems.

## 7 Conclusions and perspectives

### 7.1 A paradigm under construction

Futile recanalization stands as the central unresolved challenge of the mechanical thrombectomy era. That approximately half of patients who achieve successful macrovascular reperfusion nevertheless fail to regain functional independence reveals a fundamental truth: opening a vessel is a necessary but insufficient condition for neurological recovery. The essence of this failure lies in the dissociation between macrovascular patency and effective tissue-level reperfusion—a gap driven by microvascular no-reflow, early arterial reocclusion, collateral insufficiency, and reperfusion-mediated injury, each interacting within a self-reinforcing pathological cascade.

The tissue clock concept represents the most important conceptual advance in addressing this gap. By reframing treatment eligibility from uniform time thresholds to individualized tissue viability assessment, it has already transformed clinical practice: the DAWN and DEFUSE 3 trials demonstrated that imaging-guided selection enables safe and effective thrombectomy well beyond conventional time windows, establishing the primacy of tissue status over elapsed time (6, 7). This paradigm shift—from “time is brain” to “tissue status determines prognosis”—has opened new possibilities for precision stroke treatment.

### 7.2 Persistent challenges

Translating the tissue clock concept from a trial-validated principle to a universally implemented clinical standard requires overcoming obstacles at multiple levels. At the technical level, imaging biomarkers—including ASPECTS, perfusion-derived core and penumbra volumes, collateral grading, and net water uptake—remain inadequately standardized across devices, software platforms, and institutions. Automated tools show promise for reducing variability but have not yet achieved the reliability required for standalone clinical deployment. At the scientific level, the complex interactions among FR mechanisms mean that single-target interventions are unlikely to be sufficient; mechanism-based research that characterizes the dominant pathological process in individual patients and guides targeted combination therapy remains in its early stages. At the level of clinical translation, most prediction models—whether traditional scores or machine learning algorithms—lack prospective validation in diverse, multicenter populations, and the interpretability of AI-based models remains a barrier to clinical adoption. At the systems level, the advanced imaging, computational infrastructure, and procedural expertise required for tissue clock-guided treatment are unevenly distributed globally, creating a risk that precision stroke care will exacerbate rather than reduce health disparities.

### 7.3 Priority directions

Addressing these challenges will require coordinated progress across several domains. Standardization and quality governance—including unified imaging acquisition protocols, harmonized post-processing algorithms, and consensus reporting frameworks—are essential prerequisites for multicenter research and model generalization. The integration of artificial intelligence into clinical workflows must advance beyond isolated prediction tools toward comprehensive, real-time decision support that is transparent, rigorously validated, and embedded seamlessly in the treatment chain. Combination treatment strategies, guided by individual mechanism profiling, offer the potential to address the multifactorial nature of FR more effectively than any single intervention. Neuroprotective strategies, which have failed repeatedly in previous decades, may warrant re-evaluation in the context of mechanical thrombectomy, where tissue clock-guided patient selection and precise perioperative timing could overcome the limitations that undermined earlier trials. Finally, equitable access to precision stroke care—through telemedicine networks, simplified imaging protocols, and investment in operator training across geographic and economic settings—must be prioritized as a matter of both clinical effectiveness and ethical responsibility.

### 7.4 Clinical practice framework

For the practicing clinician facing an individual stroke patient, the principles emerging from this body of evidence can be distilled into three guiding questions. First, what is the tissue clock status? Integrating ASPECTS, core volume, collateral grading (using validated scales such as the ASITN/SIR or multiphase CTA systems), and edema severity (NWU) provides a multidimensional assessment of tissue viability that is more informative than elapsed time alone. Second, what is the individualized FR risk? Clinical scores, imaging biomarkers, and—where available—computational prediction models enable pre-treatment risk stratification that can inform shared decision-making with patients and families, ensuring realistic expectations. Third, how can the intervention be optimized? Procedural quality—targeting first-pass complete recanalization with appropriate catheter technology (balloon guide catheter, optimized device selection), careful hemodynamic management, rational adjunctive pharmacotherapy (intra-arterial thrombolysis for microvascular residual thrombi, etiology-guided antiplatelet therapy for ICAS-related occlusions), and structured post-thrombectomy neurocritical care—collectively modulate the transition from vessel patency to tissue recovery.

### 7.5 Concluding perspective

Futile recanalization should not be understood simply as a failure of recanalization, but as a failure of translation—from arterial reopening to tissue salvage, network preservation, and lasting clinical benefit. The tissue clock offers a practical lens for narrowing this gap because it links treatment timing to the questions that matter most at the bedside: how much brain remains salvageable, whether reperfusion can be effectively delivered at the tissue level, and how likely that tissue rescue is to produce functional benefit given infarct location and biological recovery reserve. Going forward, reducing FR will require moving beyond reperfusion itself toward integrating precise patient selection, high-quality thrombectomy, mechanism-guided adjunctive therapy, and vigilant post-thrombectomy care across the stroke treatment pathway.
